# Depression among teachers: a Swedish register-based study

**DOI:** 10.1186/s12889-022-12758-0

**Published:** 2022-02-19

**Authors:** Erika Johansson, Daniel Falkstedt, Melody Almroth

**Affiliations:** Institute of Environmental Medicine, Karolinska Institutet, Solnavägen 4, 113 65, floor 10, Stockholm, Sweden

**Keywords:** Depression, Gender differences, Mental health, Teachers, Work stress

## Abstract

**Background:**

Some studies have suggested that teachers are particularly at risk for mental health problems but the research in this area has not been conclusive. This study aims to compare the risk of being diagnosed with depression for different types of teachers in Sweden, both with each other and with the rest of the population, using register data.

**Methods:**

Just over 3 million individuals age 30-60 were included of which 256,166 were teachers. The exposure variable was the occupation held in 2005 and the outcome was any diagnosis of depression during the follow up period of 2006 to 2016. The data was analyzed using Cox proportional hazard regression.

**Results:**

Around 5 % of women and 3 % of men developed depression during the follow up. For women, teachers had a decreased risk of developing depression compared to non-teachers, but this association was no longer present after adjusting for common covariates including education. For men, teachers had an increased risk of depression both before and after adjustment (HR 1.27 95% CI 1.22-1.32). When comparing different kinds of teachers to university teachers, several types of teachers including primary and special education teachers had an increased risk among men while primary and secondary teachers had a decreased risk among women.

**Conclusion:**

The increased risk of depression in male teachers is a result that requires further exploration in terms of occupational differences between male and female teachers.

**Supplementary Information:**

The online version contains supplementary material available at 10.1186/s12889-022-12758-0.

## Background

Depression is a major contributor to health loss worldwide. In 2015 alone depression was estimated to have caused over 50 million Years Lost due to Disability, YDL [1]. A person who has once suffered from depression is at an increased risk for several physical ailments as well as an increased risk for premature death [2]. Work stress has been identified as a risk factor for depression, and one occupation that seems to be particularly vulnerable is teachers [2–4]. This may be due to teachers having very high quantitative and emotional demands [3, 4]. Teachers in Sweden may be at particularly high risk due to reorganization, decentralization, and privatization of the schools in the last decades, which has been said to have increased the workload for teachers [5].

Several studies have investigated the mental health of teachers. In a sample of English primary teachers, Titheradge et al. [6] found that compared to other professionals, teachers were more likely to report clinically significant psychological distress, though they reported lower levels compared to patients diagnosed with depression. Similarly, a study of teachers in Sweden found that 15% scored high on at least two out of the three burnout dimensions; exhaustion, cynicism and low professional efficacy [3]. A register-based study conducted in Denmark found that teaching, together with health professions, were the jobs associated with the highest risk for women to develop affective or stress-related disorders. Men working in preprimary education have also been found to have an elevated risk for developing these disorders [4].

On the other hand, in a critical review of 28 studies published in the field between 1986 and 2014, including the Danish study mentioned above, Van Droogenbroeck & Spruyt [7] found no conclusive evidence indicating that teachers have worse mental health than other professions. They did, however, find many methodological limitations in the existing literature. For example, different studies had defined mental health/illness differently resulting in many different instruments being used for measuring. Also, the teacher samples were often not compared with a representative sample of other occupations, but rather compared with data collected in another study, maybe several years earlier and/or in an entirely different context. Van Droogenbroeck & Spruyt [7] also found many of the earlier studies lacking when it came to defining which kinds of teachers they studied (for example, teachers in special education or university teachers) and also defining exactly which occupations they compared the teachers with. Their suggestion is that future studies use the International Standard Classification of Occupations (ISCO) for this purpose.

Van Droogenbroeck & Spruyt [7] later tried to address the above-mentioned limitations in a study of their own. In that study they compared data from Belgian teachers with data from 31 other occupations. Their result showed that teachers did not have worse mental health than people in other occupations and that people with less education were more likely to suffer from mental illness than teachers. They also did not find any difference in mental illness between different types of teachers.

The causes of depression are complex and like many other psychiatric disorders it is believed that both biological, social, and psychological factors are at play in its development. Besides work stress there are several other common risk factors, for example being a woman, being older, being a migrant, having less education, having a previous psychiatric diagnosis, or working 75% or less [2, 8–13].

Considering the disparate conclusions and methodological limitations of earlier research, the aim of this study is to compare the risk of being diagnosed with depression for teachers compared to non-teachers in Sweden, and among different types of teachers using a large nationwide sample based on register data with clearly defined variables (i.e., of depression and occupation) and accounting for potentially important confounders.

## Methods

### Study population and design

The study population is based on the SWIP cohort (Swedish Work, Illness, and labor market Participation). This is a register-based cohort linking information from the total population register, the LISA register (Longitudinal Integrated Database for Health Insurance and Labor Market Studies) and the national patient registers. These registers are presented in more detail elsewhere [14–16]. The linkage between registers was carried out by Statistics Sweden (Statistiska Centralbyrån) meaning that the data records are deidentified and cannot be traced back to a specific individual. The study was approved by the Regional Ethics Review Board in Stockholm (reference number 2017/1224-31 and 2018/1675-32).

The initial cohort includes all individuals between 16 and 65 registered in Sweden in 2005 (around 5.4 million). However, this study focuses on adults between 30 and 60 years in 2005 with occupational information, a total of 3,017,955 people. The cohort is further described elsewhere [17]. The age range 30-60 was chosen in order to reach people well established in the labor market, that is, people who are more likely to have finished their education and settled into an occupation, as well as those more likely to be stable in their chosen occupation during the follow-up period. Some of the analyses in this study are, however, done on a sub-population of teachers only. The teacher sub-group included a total of 256,166 people.

### Measures

#### Exposure

The exposure variable was occupation held in 2005, based on information from the LISA register. Type of occupation was defined by the Swedish version of the International Standard Classification of Occupations (ISCO) codes. The four-digit ISCO codes from the 1996 version were used, since those were the codes used at the time of exposure [18]. Though there are many types of professions that do similar work as teachers in the Swedish school system, like teacher’s assistants, youth recreation leaders or childcare workers, the focus for this study are on those directly involved in teaching and planning teaching activities. In Table 1 the different types of teachers included are listed with their respective ISCO code.

**Table 1:** Different types of teachers

ISCO code^a^	Type of teacher
2310	College, university and higher education teachers
2321	Secondary education teachers - academic subjects
2322	Secondary education teachers - vocational subjects
2323	Secondary education teachers - teachers in artistic and practical subject
2330	Primary education teachers
2340	Special education teachers
2351	Education method specialists and related professionals
2359	Teaching professional not elsewhere classified
3310	Pre-primary education teachers
3320	Other teaching associated professionals

^a ^Indicates the Swedish SSYK96 code which corresponds to the International Standard Classification of Occupations code

College, university and higher education teachers work with adult students and scientists at university level. Secondary education teachers work with teenagers between 16 and 19 years of age in upper-secondary school, which is voluntary in Sweden though almost all attend. Primary education teachers are mainly found in the compulsory schools. In Sweden, primary school starts at the age of six, beginning with a year of preschool class followed by grades one to nine. Special education teachers refer to teachers working with physically or mentally disabled students and/or students who have reading and writing difficulties. Education method specialists and related professionals work with developing teaching methods, act as advisors in pedagogical and educational questions, evaluate the curriculum and policies etc. Teaching professional not elsewhere classified can, for example, work in companies that specialize in education, for example giving classes in computer science or leadership. They can also work as art or music teachers. Teachers in Swedish for immigrants (SFI) are also included in this category. Preprimary teachers work with children between 1 to 5 years of age in preschools, which are voluntary but low cost, and the majority of children attend. This category also includes after-school teachers [Fritidspedagoger] who often work in recreation centers [Fritidshem] which are open before and after school hours for children between six and thirteen. The last category, other teaching associated professionals, are similar to the teaching professionals not elsewhere classified mentioned above, mainly working outside the formal education system giving recreational classes in languages, crafts etc. or giving lessons in driving or flying [18, 19].

#### Outcome

The outcome variable was any diagnosis of depression in in- or out-patient registers between 2006 and 2016. In the Swedish health care system, depression is diagnosed by a health professional such as a doctor or therapist using the criteria found in the Swedish version of International Statistical Classification of Diseases and Related Health Problems, the ICD-10-SE. Depression was defined by the presence of any of the ICD codes starting with F32 or F33 [20, 21]. It is important to note, however, that these patient registers only include specialist health care and not patients in primary care or patients that have only been treated by health care professionals other than doctors [15, 22].

#### Covariates

Several covariates that have been identified in earlier research as important risk factors for depression have also been considered.

##### Birth year and sex

Information about birth year and sex were taken from the population register [14]. For descriptive analyses, age was divided into three categories: those born between 1945 and 1955 (oldest), those born 1956-1965 (middle) and those born 1966-1975 (youngest). Sex was coded as men and women respectively.

##### Percent of employment, highest achieved education and birth country

Information about percent of employment, highest achieved education and birth country were taken from the LISA register from 2005. Percent of employment was divided into four categories; those working 25% or less, those working between 26 and 50%, those working 51 to 75% and those working between 76 to 100%.

The education variable was dichotomized in order to reflect whether an individual had more or less than 15 years of education, which corresponds to compulsory school (9 years) plus upper-secondary school (3 years), plus a university education (3 years for a bachelor’s degree). The birth country variable was dichotomized as those born in Sweden and those born outside of Sweden.

##### Psychiatric diagnosis prior to baseline

Information about psychiatric diagnosis prior to baseline was taken from the in-patient register, which has information on psychiatric diagnoses starting in 1973. Psychiatric diagnoses were defined as having any diagnosis with ICD-10 codes between F00 and F99 (or the corresponding ICD-8 or ICD-9 codes for earlier years, 295-309 and 295-316, respectively) before the year of 2005 [15, 21]. The variable was dichotomized as those with a prior psychiatric diagnosis and those without. Only inpatient data was used because outpatient data was not available and reliable prior to baseline.

### Statistical analysis

Before the analysis, a decision was made to pair four of the different teacher types into two new categories. Thus, secondary education teachers in vocational subjects (2322) and secondary education teachers in artistic and practical subjects (2323) were made into one category, while teaching professionals not elsewhere classified (2359) and other teaching associated professionals (3020) were made into another. This was done because the original categories shared many characteristics with each other. For example, both teaching professionals not elsewhere classified (2359) and other teaching associated professionals (3020) are mainly working outside the formal school system [18]. This pairing also reduced the total number of groups, with eight categories being used in the final analysis. The only exception was when all teachers were compared to all non-teachers. Then all the different types of teachers were made into one single category.

The statistical analyses were conducted using SAS Enterprise Guide 7.1. Baseline characteristics for men and women were explored separately among teachers and non-teachers and among the different types of teachers. Cox proportional hazard regression models were built separately for men and women to estimate hazard ratios for the associations between exposure to different teaching occupations in 2005 and diagnosis of depression during the follow-up period. Model 1 shows the crude associations with no adjustments for covariates, though age is accounted for as the underlying time scale. Model 2 shows the fully adjusted associations where adjustments have been done for birth year (as a category for each birth year rather than grouped as in the descriptive analysis), birth country, highest achieved education, psychiatric diagnosis prior to baseline and percent of employment. In both models age was set as the underlying timescale and follow-up time was counted from baseline in 2005 until diagnosis of depression, emigration, death, or the end of the follow up period on December 31st, 2016, whichever came first.

Because individuals could potentially change occupational title during the follow-up period, we explored the Spearman correlation coefficients for occupational code according to each year of follow up. Additionally, we repeated the analyses after reducing the follow-up time to six (up to 2011) years rather than 11 years.

Education, while one of the most important potential confounders, is also very highly associated with occupational title. To further investigate the effect of this covariate, we stratified the analysis comparing teachers and non-teachers according to education level.

Finally, though individuals needed to have an occupational code in order to be included in the study, it is still possible that individuals could be on sick leave or unemployment. Because this could be unevenly distributed among teachers and non-teachers, we repeated the analyses after excluding those with at least 300 days spent on unemployment or sick leave during 2005, the baseline year when occupation was measured.

## Results

Among both men and women, teachers compared to non-teachers were more likely to be older, to have more than 15 years of education and to work 76-100%. Female teachers were less often born outside of Sweden, while male teachers were more often born outside of Sweden. Both male and female teachers were less likely to have a previous psychiatric diagnosis (Table 2).

**Table 2:** Covariates according to teachers compared to the non-teacher population

Covariate	Teachers	Non-teachers
	N (%)	N (%)
Men		
Age		
30–40	24,102 (33.16)	479,004 (33.83)
41-50	20,291 (27.92)	462,428 (32.66)
51–60	28,287 (38.92)	474,529 (33.51)
Birth country		
Sweden	63,045 (86.75)	1,252,942 (88.50)
Other	9626 (13.25)	162,812 (11.50)
Education		
≤ 15	24,988 (34.48)	1,170,563 (82.89)
> 15	47,492 (65.52)	241,607 (17.11)
Previous psychiatric diagnosis		
Yes	2842 (3.91)	62,331 (4.41)
No	69,754 (96.09)	1,352,080 (95.59)
Percent of employment		
≤ 25	10,511 (14.46)	461,653 (32.60)
26–50	4375 (6.02)	22,226 (1.57)
51–75	3708 (5.10)	19,711 (1.39)
76–100	54,086 (74.42)	912,371 (64.43)
Women		
Age		
30–40	60,434 (32.94)	434,124 (32.26)
41–50	56,078 (30.56)	443,058 (32.92)
51–60	66,974 (36.50)	468,653 (34.82)
Birth country		
Sweden	165,979 (90.45)	1,169,590 (86.91)
Other	17,502 (9.54)	176,114 (13.09)
Education		
≤ 15	74,435 (40.60)	1,083,994 (80.66)
> 15	108,908 (59.40)	259,879 (19.34)
Previous psychiatric diagnosis		
Yes	7159 (3.90)	66,598 (4.95)
No	176,203 (96.10)	1,277,992 (95.05)
Percent of employment		
≤ 25	30,479 (16.61)	317,750 (23.61)
26–50	13,047 (7.11)	81,639 (6.07)
51–75	22,411 (12.21)	171,398 (12.74)
76–100	117,549 (64.06)	775,048 (57.59)

Table 3 shows the characteristics for the different types of teachers. Among both men and women, the group, “college, university and higher education teachers” was the type of teacher with most people in the 30-40 age group, the highest degree of foreign-born members, the highest share of people with over 15 years of education, the highest share of people working 76-100%, and the least likely to have a previous psychiatric diagnosis.

**Table 3:** Covariates according to type of teaching occupation

Covariate	1	2	3	4	5	6	7	8
	%	%	%	%	%	%	%	%
Men								
Age								
30–40	40.81	27.99	24.43	37.45	17.25	33.85	29.21	39.63
41–50	27.61	25.41	30.65	25.03	23.26	30.77	31.75	32.97
51–60	31.58	46.60	44.92	37.53	59.48	35.38	39.04	27.40
Birth country								
Sweden	79.35	90.08	91.53	85.56	92.45	92.62	87.79	90.27
Other	20.65	9.92	8.47	14.44	7.55	7.38	12.21	9.73
Education								
≤ 15	5.02	15.93	61.29	23.11	29.09	62.15	71.84	73.31
> 15	94.98	84.07	38.71	76.89	70.91	37.85	28.16	26.69
Previous psychiatric								
Yes	2.49	3.86	4.33	4.30	4.25	3.40	4.46	4.94
No	97.51	96.14	95.67	95.70	95.75	96.60	95.54	95.06
Percent of employment								
≤ 25	5.18	13.37	11.01	13.95	9.53	24.62	49.64	12.66
26–50	7.62	4.60	6.87	5.95	4.29	4.00	4.28	4.57
51–75	3.69	4.96	7.57	5.44	3.30	3.38	2.76	5.37
76–100	83.51	77.08	74.55	74.66	82.88	68.00	43.33	77.39
Women								
Age								
30–40	42.65	30.03	26.27	37.51	11.29	23.08	30.70	32.95
41–50	28.45	28.11	34.46	26.05	26.05	32.51	33.28	34.90
51–60	28.90	41.86	39.27	36.44	62.66	44.42	36.02	32.15
Birth country								
Sweden	80.92	89.29	92.28	89.83	94.59	92.43	87.24	92.22
Other	19.08	10.71	7.72	10.17	5.41	7.57	12.76	7.78
Education								
≤ 15	5.82	12.07	34.40	13.63	14.58	45.78	55.14	79.81
> 15	94.18	87.93	65.60	86.37	85.42	54.22	44.86	20.19
Previous psychiatric								
Yes	3.60	4.68	4.28	3.70	4.14	3.72	4.65	3.81
No	96.40	95.32	95.75	96.30	95.86	96.28	95.35	96.19
Percent of employment								
≤ 25	6.40	18.06	15.12	16.66	9.83	13.90	43.80	16.64
26–50	9.85	7.66	9.32	6.65	6.11	6.45	7.35	6.56
51–75	6.35	9.67	12.42	9.02	9.08	13.52	6.59	17.65
76–100	77.40	64.61	63.41	67.66	74.97	66.13	42.26	59.15

1. College, university and higher education teachers (2310), 2. Secondary education teachers - academic subjects (2321), 3. Secondary education teachers – vocational subjects (2322) and Secondary education teachers - teachers in artistic and practical subject (2323), 4. Primary education teachers (2330), 5. Special education teachers (2340), 6. Education method specialists and related professionals (2351), 7. Teaching professional not elsewhere classified (2359) and other teaching associated professionals (3320), 8. Preprimary education teachers (3310)

Among men, teachers were more likely to be diagnosed with depression compared to non-teachers in the unadjusted model, and this association grew stronger after adjusting for the covariates (HR1.27 CL 1.20-1.35). Among women, teachers were less likely to be diagnosed with depression compared to non-teachers in the unadjusted model. However, this difference became insignificant after controlling for the covariates (HR 0.98 CL 0.96-1.01) (Table 4).

**Table 4:** Hazard ratios and 95% confidence intervals for depression according to teaching profession and sex

	N cases depression (%)	Model 1 HR 95% (CL)	Model 2 HR 95% (CL)
**Men**			
Teachers	2848 (4)	1.21 (1.16–1.26)	1.27 (1.22–1.32)
Non-teachers	47,194 (3)	1.00	1.00
**Women**			
Teachers	8476 (5)	0.88 (0.86–0.90)	0.98 (0.96–1.01)
Non-teachers	70,548 (5)	1.00	1.000

Model 1 unadjusted with age as the underlying time scale

Model 2 is adjusted for birth year, birth country, education, previous psychiatric diagnosis, and percent of employment

The pattern of depression among the different types of teachers also varied according to sex (Table 5). For men, being a secondary education teacher in academic subjects, a secondary education teacher in a vocational subject or a secondary education teacher in an artistic or practical subject, a primary education teacher, a special education teacher, a teaching professional not elsewhere classified or other teaching associated professional or a preprimary education teacher was associated with a greater risk of being diagnosed with depression compared to being a college, university or higher education teacher in the crude model. When adjusting for covariates, significant associations only remained among primary education teachers and special education teachers compared to college, university and higher education teachers (HR 1.18, CL 1.06-1.33 and HR 1.24, CL 1.00-1.54, respectively) while teaching professionals not elsewhere classified and other teaching associated professionals had a decreased risk (HR 0.78, CL 0.66-0.92).

**Table 5:** Hazard ratios and 95% confidence intervals for depression according to type of teacher and sex

Teacher	Men			Women		
	N cases depression (%)	Model 1 HR 95% (CL)	Model 2 HR 95% (CL)	N cases depression (%)	Model 1 HR 95% (CL)	Model 2 HR 95% (CL)
1	489 (3)	1.00	1.00	579 (5)	1.00	1.00
2	350 (4)	1.25 (1.09–1.43)	1.10 (0.96–1.27)	522 (5)	1.04 (0.92–1.17)	0.90 (0.80–1.01)
3	559 (4)	1.17 (1.04–1.32)	0.98 (0.86–1.12)	697 (5)	0.95 (0.85–1.06)	0.86 (0.77–0.96)
4	850 (4)	1.39 (1.24–1.55)	1.18 (1.06–1.33)	2704 (5)	0.94 (0.86–1.03)	0.88 (0.81–0.97)
5	101 (4)	1.38 (1.11–1.71)	1.24 (1.00–1.54)	468 (4)	0.98 (0.87–1.11)	0.94 (0.83–1.06)
6	14 (4)	1.32 (0.78–2.25)	1.14 (0.67–1.95)	27 (3)	0.72 (0.49–1.05)	0.68 (0.46–1.00)
7	253 (4)	1.16 (1.00–1.35)	0.78 (0.66–0.92)	406 (5)	1.15 (1.02–1.31)	0.84 (0.74–0.96)
8	232 (4)	1.32 (1.13–1.55)	1.09 (0.92–1.29)	3073 (5)	0.95 (0.87–1.03)	0.88 (0.80–0.98)

Model 1 is unadjusted with age as the underlying time scale

Model 2 is adjusted for birth year, birth country, education, previous psychiatric diagnosis, and percent of employment

1. College, university and higher education teachers (2310), 2. Secondary education teachers - academic subjects (2321), 3. Secondary education teachers – vocational subjects (2322) and Secondary education teachers - teachers in artistic and practical subject (2323), 4. Primary education teachers (2330), 5. Special education teachers (2340), 6. Education method specialists and related professionals (2351), 7. Teaching professional not elsewhere classified (2359) and other teaching associated professionals (3320), 8. Preprimary education teachers (3310)

For women, being a teaching professional not elsewhere classified or other teaching associated professional compared to a college, university or higher education teacher was associated with an increased risk of being diagnosed with depression in the unadjusted model. When adjusting for the covariates, secondary education teachers in vocational subjects and secondary education teachers in artistic and practical subjects, primary education teachers, teaching professional not elsewhere classified and other teaching associated professionals, and preprimary education teachers all had a decreased risk of depression compared to college, university and higher education teachers (HR 0.86, CL 0.77-0.96; HR 0.88, CL 0.81-0.97; HR 0.84, CL 0.74-0.96; HR 0.88, CL 0.80-0.98, respectively).

The covariates most to least associated with depression were previous psychiatric diagnosis, birth year, percent of employment, birth country, and education. However, the variables with the most influence on estimates of the relationship between teaching and depression were education, previous psychiatric diagnosis, and percent of employment (not shown).

Assessment of the correlation of occupational codes throughout the follow-up period indicated that the correlation tended to decrease when more years had passed, but the lowest correlation coefficient (between 2006 and 2016) was 0.71, indicating that individuals were quite consistent in their occupational titles over time (Table S1). When reducing the follow-up period to 6 years rather than 11, results were consistent and showed the same pattern as the main models (not shown).

When stratifying comparisons of teachers and non-teachers by education, adjusted models showed little difference in association (Table S2).

After excluding individuals on long term sick leave and unemployment during 2005, differences in adjusted associations were negligible, indicating that any major differences between teachers and non-teachers and the different teaching groups were accounted for when adjusting for confounders in the main analysis.

## Discussion

This register-based study of the association between teaching professions and depression found that among men, teachers were more likely to be diagnosed with depression compared to non-teachers after adjusting for covariates. For women, teachers were less likely to become depressed during the follow-up, but this association was not present after adjusting for covariates. The risk of being diagnosed with depression differed for different types of teachers and by sex. For men, an increased risk was found for primary and special education teachers compared to university teachers after adjustment, while teachers outside of the school context showed a decreased risk. For women, specialized secondary school teachers, primary school teachers, preschool teachers and teachers outside of the of the school context all showed a decreased risk compared to university teachers in adjusted models.

Several previous studies have identified teachers as a particularly high-risk group for mental health problems [3–5]. Wieclaw et al. [4], however, found both male and female teachers to be at higher risk of affective and stress disorders compared to other occupations, and Van Droogenbroeck & Spruyt [7] did not find differences in the risk of developing depression between different types of teachers.

Several important methodological differences should be noted, including rather small sample sizes [3, 6], use of cross-sectional data [7], relying on self-reported mental health scales sometimes measuring multiple dimensions of mental health or other mental health problems rather than diagnosis of depression [4, 7] and not stratifying by sex [7]. Some of these methodological limitations may lead to various biases or difficulties in interpretations.

Choice of comparison group is also an important aspect to consider. Both Van Droogenbroeck & Spruyt [7] and Wieclaw et al. [4] compared teachers with specific other occupations while in the present study teachers were compared to all non-teachers. On one hand, it is important to see how teachers compare to the general working population rather than specific other populations. On the other hand, these associations can be somewhat diluted by the fact that some occupations fare worse than teachers. Repeating our analyses after excluding those on long term sick leave or unemployment at baseline was an attempt to address potential differences in health among teachers and non-teachers.

An important finding of the present study is that female teachers actually had a reduced risk of developing depression, which was attenuated to no association after adjusting for education and other covariates. In other words, the observed protective effect of teaching on depression for women was explained by their higher level of education and other sociodemographic factors. Male teachers, however, had an increased risk of developing depression both before and after adjustment. It has been suggested that higher education tends to protect women from depression to a higher degree than men because women’s relative social disadvantage makes education more important for improving well-being [12].

Higher education tends to be related to more positive occupational and health outcomes. Given that teachers are more highly educated than the general population, one could reasonably suspect that teachers may have a lower risk of depression. However, that men had an increased risk of depression even after adjusting for important covariates, including education, indicates that this increased risk is due to something else experienced by male teachers.

Other studies have also shown similar patterns between men and women in other occupations. For example, a study of British doctors found that male doctors experienced more anxiety and depression than the average British male while female doctors had significantly better mental health than the normative population [23]. Cobos-Sanchiz, Del-Pino-Espejo, Sánchez-Tovar, and Matud MP [24] found that the association between job events and job changes and psychological distress was stronger for men than for women. This indicates that men and women are affected differently by aspects of their occupations.

The etiology of depression may differ between men and women in important ways. As noted by Kendler and Gardner [25] in their study of depression in dizygotic twins, depression in women could, to a higher degree, be explained by personality and problems in interpersonal relationships while for men the leading causes seemed to be externalizing psychopathology, prior depression, and specific stressors like failure at work or providing for the family.

Men and women’s care seeking for depression may also differ in important ways. For example, women tend to seek help for mental health issues to a higher degree than men [26]. Women may seek help earlier than men and may thus be treated in primary care where they would not be identified in the hospital and specialist care registers. Men may wait longer to seek help, and when they do, they could potentially have more problems and, therefore, be treated in specialist care to a higher degree. It has been suggested, however, that men in female dominated occupations may be more likely to seek help due to greater mental health awareness in these environments [27].

The observed differences between men and women in the comparison of different teaching occupations is an interesting result that deserves further exploration. That other types of teachers compared to university teachers tended to have a higher risk among men and a lower risk among women may represent particular gender patterns within these occupations. Teaching occupations outside of higher education may be seen as less prestigious, and these jobs are also clearly female dominated. It may be that men are less satisfied in these positions and that this affects their mental health more than women.

Another possibility may be that specific factors of these work environments differ between men and women. A direction for further research could be to explore potentially mediating factors in the association between teaching occupations and depression among men. Factors of the work environment such as violence, including verbal and physical violence, which previous studies have found to be more common among special education teachers [28], may be an important aspect in understanding why male special education and primary school teachers had a higher risk of depression than university teachers.

When comparing teachers outside of the traditional school environment, the same pattern was seen for both men and women where an increased risk of depression was found before adjustment and a decreased risk after adjustment. This was primarily explained by adjusting for percent of employment, which this particular group was much more likely to work less than 75%. In this case, percent of employment can be seen as a potential confounder or a mediator. Individuals with mental health problems may have difficulty working full time and may thus choose a part time occupation representing a possible confounding or selection effect. On the other hand, part time work may be a consequence of certain occupations, leading to a causal mediation effect. In either case, part-time employment is an important aspect of why individuals in this group appeared to have a higher risk of depression before adjustment.

### Strengths and limitations

This study has several strengths. It is based on a large study sample with a wide geographic spread (including all of Sweden). It is also based on register data which minimizes the risk for selection biases [29], and the study sample has been followed for a period of over 10 years. It is not without weaknesses, however, one being the lack of information about patients being treated for depression in primary care. This means that only more severe cases of depression were identified. Recent data has suggested antidepressant use in the 30-60 age group in Sweden to be around 12% [30], indicating an underestimation in our results. However, antidepressants are often prescribed for anxiety or other illnesses besides depression. Focusing on severe diagnosed depression reduces the risk of misclassification in this regard.

Occupational title held in 2005 was the exposure investigated, and we did not account for how that exposure may have changed during the follow-up period. However, restricting the population to those over 30 years of age at baseline was a deliberate decision to include those who were more likely to be stable in a career. Furthermore, we found the correlation of occupational title to be quite high over the follow-up period and found similar results when using a reduced follow-up period.

Although our study did control for several other known risk factors for depression, there is always a risk for unmeasured confounding. Some personality factors, for example, could be important in these associations. One such factor is over-commitment to work, which may be more common among teachers and may also be related to mental health problems [4]. Additionally, our study population is a closed cohort who were registered in Sweden in 2005. We therefore start our baseline in 2005 rather than the moment where a person enters a teaching profession. When we adjust for psychiatric diagnoses prior to baseline, this may include some cases that were working as teachers prior to baseline as well, indicating a potential overadjustment. However, the implication of not adjusting for psychiatric diagnoses would likely be more misleading. On a related note, outpatient data was not available prior to baseline, and inpatient psychiatric data was only available starting in the 1970s, indicating that some of the older individuals did not have available information from their early life.

Finally, Sweden is a rather unique context when it comes to educational policies and specific changes that have taken place in the last several decades. This includes a very rapid decentralization of the school system, as well as privatization of schools, and the emergence of publicly funded but privately run schools which are allowed to operate for profit [31, 32]. These specific aspects may affect the generalizability of these results to other educational contexts.

## Conclusions

That male teachers had an increased risk for depression compared to the general working population is a finding that deserves further exploration. Depression among teachers affects not only themselves, but also the students they teach and their families. It is therefore important to further understand what specific factors may be leading to an increased risk of depression among male teachers. Future studies should further consider differences between male and female teachers in terms of work environment and other factors related to teaching occupations.

## Supplementary Information

**Additional file 1: Table S1.** Spearman correlation of occupational code (SSYK code) during the follow-up period. **Table S2.** Hazard ratios and 95% confidence intervals for depression according to teaching profession and sex, stratified by education level.

## Data Availability

The data that support the findings of this study are based on information from the national Swedish registers available from Statistics Sweden (SCB.se). as third party holders, we do not have the rights to share the data publicly. More information is available from Statistics Sweden or the corresponding author.
