# Supplementary material for: Depression among teachers: a Swedish register-based study
Source: BMC Public Health. 2022 Feb 19;22:355. doi: 10.1186/s12889-022-12758-0 (PMC8857808; doi:10.1186/s12889-022-12758-0)
Supplement: Supplementary file 1 — Additional file 1: Table S1. Spearman correlation of occupational code (SSYK code) during the follow-up period. Table S2. Hazard ratios and 95% confidence intervals for depression according to teaching profession and sex, stratified by education level. [file 12889_2022_12758_MOESM1_ESM.docx]

**Table S1**

*Spearman correlation of occupational code (SSYK code) during the follow-up period*

|  | **2006** | **2007** | **2008** | **2009** | **2010** | **2011** | **2012** | **2013** | **2014** | **2015** | **2016** |
| --- | --- | --- | --- | --- | --- | --- | --- | --- | --- | --- | --- |
| **2006** | 1 |  |  |  |  |  |  |  |  |  |  |
| **2007** | 0.95 | 1 |  |  |  |  |  |  |  |  |  |
| **2008** | 0.91 | 0.95 | 1 |  |  |  |  |  |  |  |  |
| **2009** | 0.89 | 0.92 | 0.96 | 1 |  |  |  |  |  |  |  |
| **2010** | 0.86 | 0.89 | 0.92 | 0.95 | 1 |  |  |  |  |  |  |
| **2011** | 0.84 | 0.86 | 0.90 | 0.92 | 0.96 | 1 |  |  |  |  |  |
| **2012** | 0.82 | 0.84 | 0.87 | 0.90 | 0.93 | 0.96 | 1 |  |  |  |  |
| **2013** | 0.81 | 0.83 | 0.86 | 0.88 | 0.91 | 0.94 | 0.97 | 1 |  |  |  |
| **2014** | 0.74 | 0.75 | 0.77 | 0.78 | 0.79 | 0.81 | 0.83 | 0.85 | 1 |  |  |
| **2015** | 0.73 | 0.74 | 0.76 | 0.77 | 0.78 | 0.80 | 0.81 | 0.83 | 0.97 | 1 |  |
| **2016** | 0.71 | 0.73 | 0.74 | 0.75 | 0.76 | 0.78 | 0.79 | 0.81 | 0.94 | 0.96 | 1 |

**Table S2**

*Hazard ratios and 95% confidence intervals for depression according to teaching profession and sex*

|  | | **Men** |  | **Women** |  |
| --- | --- | --- | --- | --- | --- |
|  | | **N cases depression (%)** | **HR 95% (CL)** | **N cases depression (%)** | **HR 95% (CL)** |
| **Education <15** | |  |  |  |  |
| Teachers | | 1127 (5) | 1.27 (1.20-1.35) |  | 0.96 (0.93-1.00) |
| Non teachers | | 40479 (3) | 1.00 | 3559 (5) | 1.000 |
| **Education >15** |  | |  | 57974 (5) |  |
| Teachers | | 1717 (4) | 1.26 (1.19-1.33) | 4916 (5) | 0.99 (0.96-1.02) |
| Non teachers | | 6610 (3) | 1.00 | 12455 (5) | 1.00 |

*Note.* Models are adjusted for birth year, birth country, previous psychiatric diagnosis, and percent of employment.
